# IgG4-related hypertrophic pachymeningitis with ANCA-positivity: A case series report and literature review

**DOI:** 10.3389/fneur.2022.986694

**Published:** 2022-09-15

**Authors:** Cheng Xia, Ping Li

**Affiliations:** ^1^Department of Neurology, General Hospital of Northern Theater Command, Shenyang, China; ^2^Department of Rheumatology, General Hospital of Northern Theater Command, Shenyang, China

**Keywords:** hypertrophic pachymeningitis, IgG4-related disease, antineutrophil cytoplasmic antibody, ANCA-associated vasculitis, IgG4

## Abstract

**Background:**

Hypertrophic pachymeningitis (HP) is a rare inflammatory disorder characterized by local or diffuse thickening of the intracranial or spinal dura mater. The most frequent cause of HP is antineutrophil cytoplasmic antibodies (ANCA), followed by IgG4. However, few cases of IgG4-HP coexpressing ANCA have been reported. Herein, we present three cases of IgG4-HP coexpressing ANCA and review the relevant literature to document the overlap of these two HP causes as a potential clinical pattern.

**Methods:**

We retrospectively analyzed three patients with IgG4-HP coexpressing ANCA in our center and consulted the PubMed database to find other relevant cases reported in English from 1976 to April 2022. We used the following keywords: pachymeningitis, meningitis, dura, antineutrophil cytoplasmic antibody, myeloperoxidase, and proteinase-3. We analyzed the clinical, serological, radiological, and pathological characteristics of the obtained cases based on the ACR and Chapel Hill criteria and the exponential moving average (EMA) algorism for ANCA-associated vasculitis (AAV) and the IgG4-RD Comprehensive Diagnostic Criteria.

**Results:**

We analyzed a total of 10 cases: seven literature reports and our three patients (52- and 61-year-old women and a 65-year-old man). The IgG4-related disease (IgG4-RD) diagnoses were definitive in four cases, and probable and possible in three cases. Eight patients had ANCA against myeloperoxidase (MPO), and two had ANCA against proteinase-3 (PR3). Two patients had both IgG4-RD and AAV, while the others only had ANCA seropositivity without additional clinical or pathological markers of AAV.

**Conclusion:**

With regard to HP, we reconfirmed the existence of the IgG4-RD and AAV overlap syndrome. Meanwhile, our review does not support the hypothesis that ANCA positivity in IgG4-RD results from an excessive B-cell response. We speculate that IgG4-RD and AAV have similar or associated pathogeneses, although uncovering the role of IgG4 and ANCA in these pathophysiological processes requires further investigation.

## Background

Hypertrophic pachymeningitis (HP) is a group of rare disorders characterized by local or diffuse thickening of the intracranial or spinal dura mater causing intracranial hypertension, cranial nerve palsy, or spinal cord dysfunction. Headache is the most common initial symptom of HP (1). The main pathological signs of HP include interstitial fibrosis and infiltration of inflammatory cells, mainly lymphocytes. The identifiable causes of HP are heterogeneous and include infections (i.e., *Mycobacterium tuberculosis*, fungi, or *Borrelia burgdorferi*), inflammatory diseases (i.e., IgG4-related disease (IgG4-RD), sarcoidosis, Sjogren's syndrome, rheumatoid arthritis, or Wegener's granulomatosis) (2). A nationwide investigation in Japan revealed that the most frequent cause of HP was antineutrophil cytoplasmic antibodies (ANCA) (30.2%), followed by IgG4 (8.8%) (1). ANCA-related HP comprises three underlying disorders: granulomatosis with polyangiitis (GPA) (1), microscopic polyangiitis (MPA) (3), and eosinophilic GPA (EGPA) (4), which is most commonly caused by GPA (5).

The IgG4-RD is a chronic inflammatory disorder with the following pathological characteristics: lymphoplasmacytic infiltration of numerous IgG4+ cells, storiform fibrosis, and obliterative phlebitis in various organs (e.g., the salivary glands, bile ducts, thyroid glands, lungs, and pancreas) (6). The characteristic features of IgG4-RD include elevated serum IgG4 and infiltration of IgG4+ cells; however, GPA, EGPA, pulmonary sarcoidosis, and lymphoma can cause similar pathological or serological manifestations (7). Therefore, the differential diagnosis of IgG4-RD includes neoplasms, infectious diseases, and autoimmune disorders such as ANCA-associated vasculitis (AAV).

An overlap between AAV and IgG4-RD has been described in some clinical patterns, such as tubulointerstitial nephritis, periaortitis, and prevertebral fibrosis. Therefore, we hypothesized that these two diseases could also overlap in the clinical pattern of HP. Herein, we present three new cases of patients with IgG4-HP coexpressing ANCA. In addition, we review the relevant literature to identify the clinical characteristics of IgG4-HP cases coexpressing ANCA and confirm whether the overlap of IgG4-RD and AAV also exists in HP.

## Methods

### Case presentation

#### Case 1

**Figure 1:**
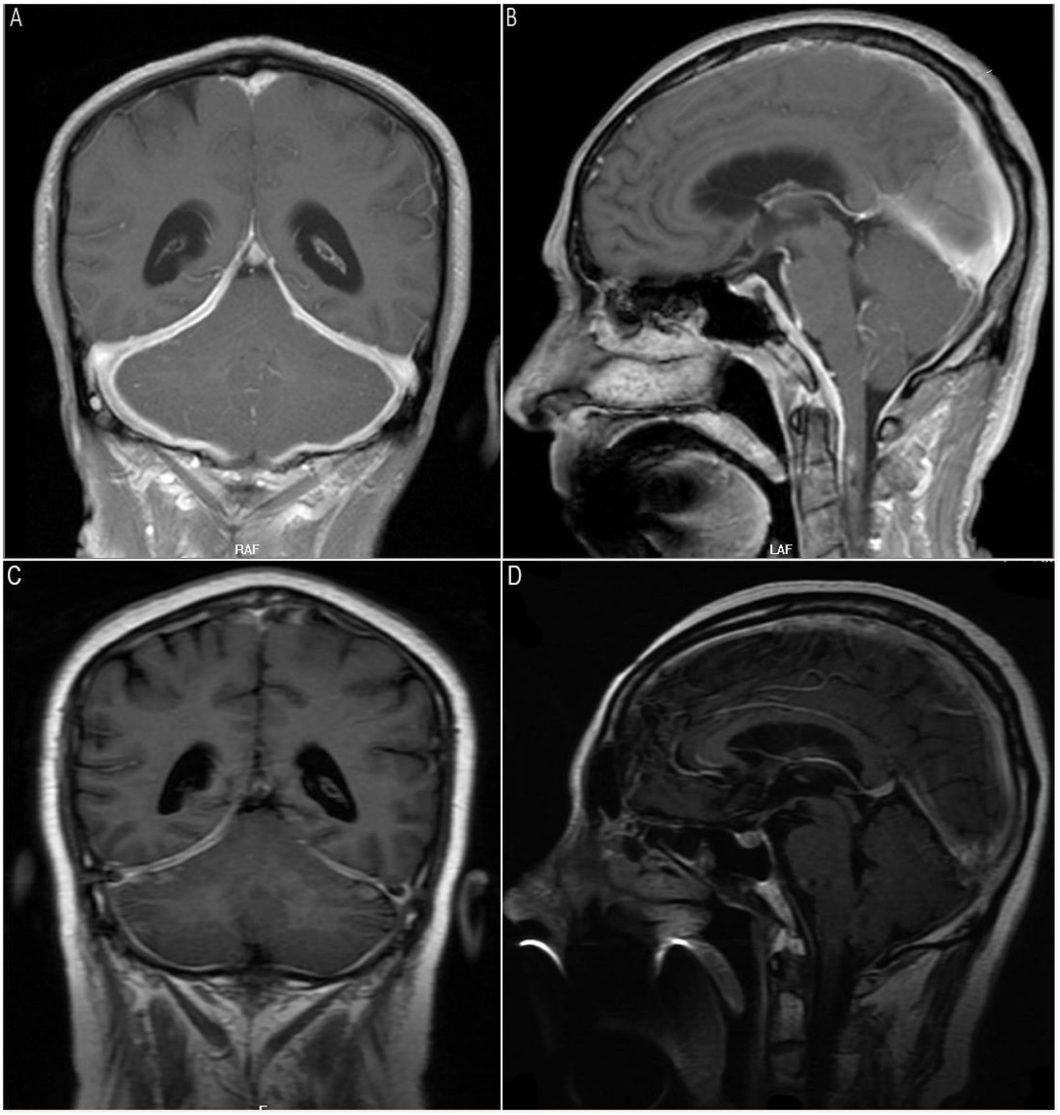
Magnetic resonance imaging (MRI) scan of the brain on admission. T1-weighted gadolinium-enhanced brain MRI revealed enhancement and thickening of dura mater predominantly in the posterior fossa **(A,B)**. Marked reduction of dura thickening and enhancement was evident at the 6-month follow-up **(C,D)**.

The first case was a 65-year-old man with paroxysmal bilateral temporal headache, a 20-kg weight loss over 3 months, hoarseness, dysphagia with paroxysmal diplopia, and intermittent fever for 1 month. A neurological examination revealed dysarthria, restricted right eye abduction, and left vocal cord paralysis. Laboratory tests yielded the following results: white blood cell count, 10,010/μl; hemoglobin, 6.9 g/dl; erythrocyte sedimentation rate (ESR), 140 mm/h; C-reactive protein (CRP), 131 mg/L. Tests for infections were negative, including blood bacteria cultures, *Mycobacterium tuberculosis* antibodies, respiratory viral antigens, human immunodeficiency virus, and fungi antigen. Rheumatologic assays were negative (such as antinuclear antibody, cyclic-citrullinated peptide IgG antibody, rheumatoid factor, anti-cardiolipin antibody, lupus anticoagulant, and angiotensin-1 converting enzyme), except for titers of serum cytoplasmic ANCA (1:32), myeloperoxidase antibodies (MPO-ANCA) (1:100) and elevated IgG4 (411 mg/dl; normal: 8–140 mg/dl), although the total IgG level was normal. Routine urinalysis was unremarkable. Cerebrospinal fluid (CSF) showed lymphocytosis (25 cells/mm^3^), and three well-defined oligoclonal bands were present in both the CSF and serum. The CSF infectious disease test was negative. Chest and paranasal sinus CT found no abnormalities. Gadolinium-enhanced brain magnetic resonance imaging (MRI) revealed enhanced and thickened dura mater, predominantly in the posterior fossa (Figure 1). Intravenously administered dexamethasone (10 mg/day for 5 days) markedly relieved the patient's headache, but it recurred after the oral administration of prednisolone (28 mg/day). An additional intravenous cyclophosphamide administration (0.4 g once) achieved stable improvement. On discharge, fever and headache had disappeared, and dysarthria had significantly improved. The 6-month follow-up examination showed great clinical and radiological improvement, and intravenous cyclophosphamide administration at intervals allowed to taper prednisolone. The CRP and ESR also returned to normal levels.

#### Case 2

**Figure 2:**
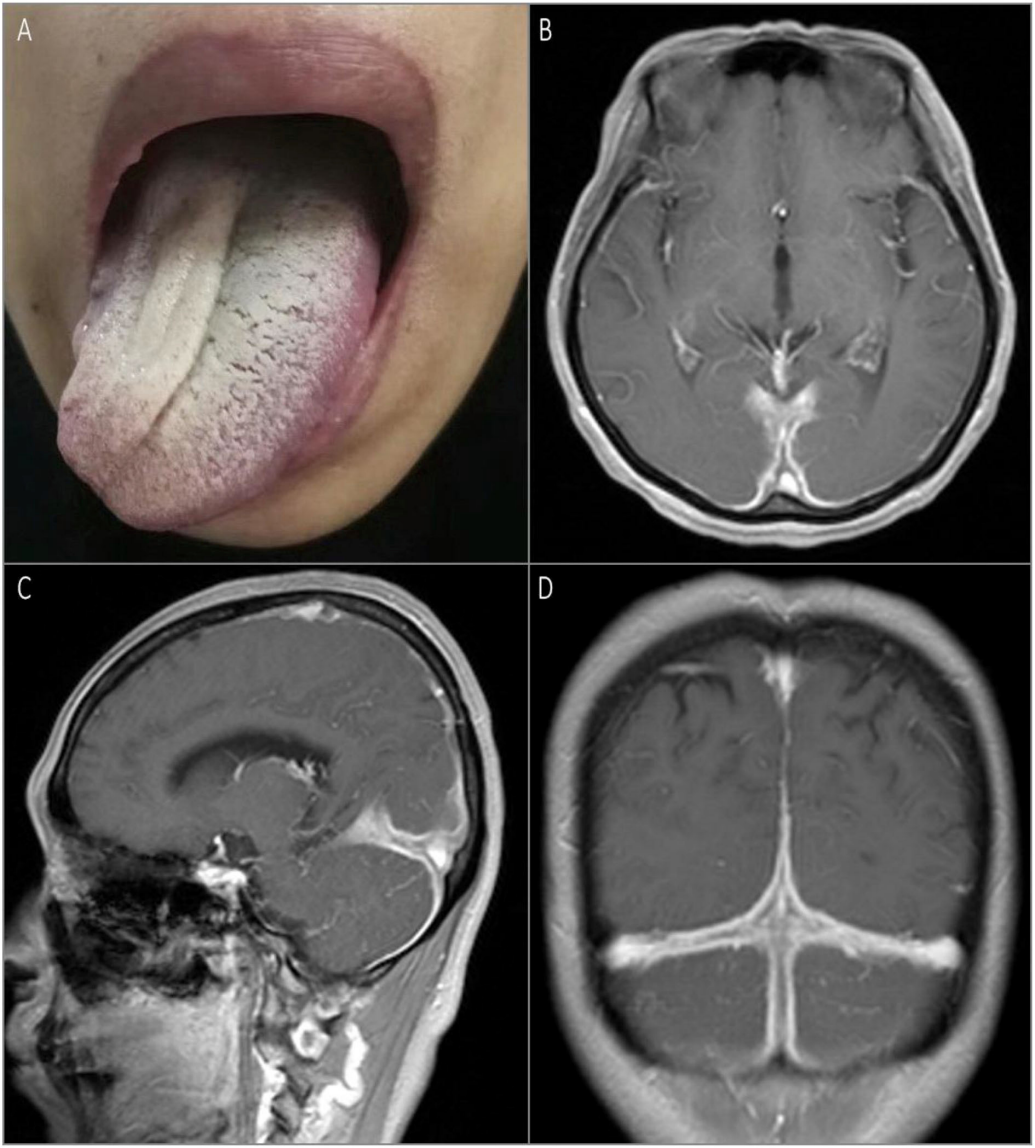
Physical examination and MRI scan of the brain on admission. Rightward tongue deviation upon protrusion and atrophy of right lingualis were observed on examination **(A)**. T1-weighted gadolinium-enhanced brain MRI showed pachymeningeal enhancement and thickening predominantly in the posterior fossa and bilateral posterior cerebral hemispheres **(B–D)**.

The second patient was a 52-year-old previously healthy woman with an ingravescent occipital headache for 6 months. The only notable neurological examination result was a right hypoglossal nerve palsy (Figure 2A). The gadolinium-enhanced brain MRI revealed pachymeningeal enhancement and thickening, predominantly in the posterior fossa and bilateral posterior cerebral hemispheres (Figures 2B–D). Infection tests were negative. Rheumatologic tests were also negative, except for the elevated rheumatoid factor (167.4 IU/ml) and CRP (87 mg/L). Urinary protein and occult blood tests were both negative. Serum levels of total IgG and IgG4 were 1,646 mg/dl (normal: <1,600 mg/dl) and 512 mg/dl, respectively, with an IgG4/IgG ratio of 31%. Serum was positive for perinuclear ANCA (titer, 1:10) and MPO-ANCA (titer, 1:100). The CSF showed lymphocytosis (33 cells/mm^3^). The CSF infectious disease test was unremarkable. Chest and paranasal sinus CT found no abnormalities. The patient received methylprednisolone pulse therapy (1 g/day, halved every 3 days until reaching 120 mg/day), followed by oral prednisone 60 mg/day, gradually reduced, and combined with oral cyclophosphamide 50 mg/day for about 3 months, with obvious relief of the headache and improvement of lingual symptoms.

#### Case 3

**Figure 3:**
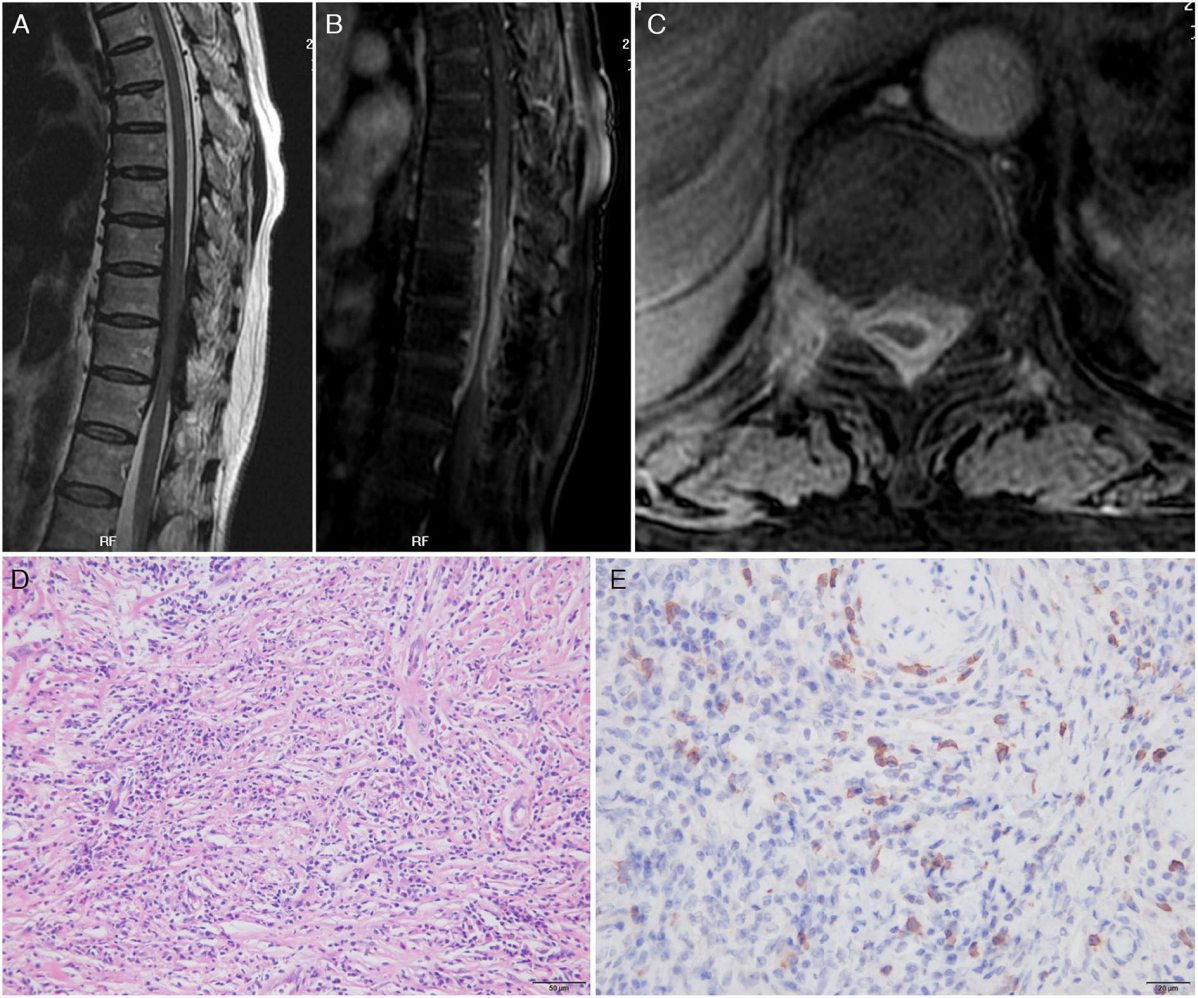
Magnetic resonance imaging scan of the thoracic spine on admission and pathology findings. Sagittal T2-weighted image **(A)** showed a low-signal intensity lesion in the anterior and posterior epidural spaces and high enhancement after gadolinium enhancement **(B)** at the thoracic canal between T7 and T11 vertebral body levels. Axial images showed that the lesion was located in the epidural space and extended to the neural foramen to the right. A fat-suppressed T1-weighted image with gadolinium enhancement demonstrated high enhancement of the mass **(C)**. Pathology findings: Hematoxylin and eosin stain of the epidural mass showed intense lymphoplasmacytic inflammatory cell infiltrate with fibrosis. Plasma cells and lymphocytes were also detected within the wall of a vessel as well as in the perivascular area **(D)**. IgG4 immunohistochemistry showed prominent IgG4+ cells within the inflammatory infiltration. Nearly more than half of the plasma cells exhibited IgG4 reactivity **(E)**.

The third patient was a 61-year-old woman admitted for weakness and stiffness in both lower limbs for about 1 year, accompanied by thoracic back pain and constipation for 8 months. The neurological examination on admission revealed paresis in the bilateral lower limbs (3 to 4/5), decreased pain and thermal sensation in the trunk and lower limbs below the T10 level, brisk bilateral knee and Achilles tendon reflexes, and bilateral extensor plantar response. The routine blood tests and inflammatory markers levels were normal. Infections assays were negative. Rheumatologic assays were negative, except for seropositivity for perinuclear ANCA (titer, 1:32), MPO-ANCA (titer, 1:100), and elevated IgG4 (441 mg/dl). Chest and paranasal sinus CT found no abnormalities. A spinal cord MRI revealed a ribbon-like thickening of the dura mater between vertebral levels T7 and T11 (which was moderately enhanced by gadolinium administration) and a compressed and flattened focal spinal cord (Figures 3A–C). Although the patient underwent emergent T7–T11 right pediculectomy and partial corpectomy for decompression and resection of the dural lesion, her neurological deficits did not improve. The CSF analysis during the operation was negative for bacteria, tuberculosis, viruses, and fungi. A broad panel of immunohistochemical markers was assayed, such as S-100, EMA, CK-P, GFAP, Vim, CD20, CD68, CD38, CD138, CD34, and Ki67. A histopathology analysis showed lymphocyte infiltration, high IgG4+ cell infiltration, and a storiform pattern of fibrosis without granulomatous changes (Figures 3D,E).

## Results: Review of the literature and our cases

We systematically searched the PubMed database for studies on humans, written in English, and published between 1976 and April 2022 using the keywords: “pachymeningitis, meningitis, dura, antineutrophil cytoplasmic antibody, myeloperoxidase, and proteinase-3.” We excluded cases described in insufficient detail. Ultimately, we analyzed 10 cases (8–14), including our three cases. The patients' ages ranged from 48 to 79 years (median 61.5 ± 3.2 years), and the male/female ratio was 1:1. Table 1 summarizes the clinical, radiological, and pathological characteristics of all patients. We re-evaluated the cases according to the Comprehensive Diagnostic Criteria (CDC) for IgG4-RD (15). Possible IgG4-RD was defined by suggestive organ involvement associated with elevated serum IgG4 levels (>135 mg/dl). We identified probable IgG4-RD by looking for classical histopathological features (i.e., dense lymphoplasmacytic infiltration, storiform fibrosis, obliterative phlebitis, and mild-to-moderate eosinophil infiltration), IgG4/IgG positive plasma cell ratio >40% and >10 IgG4+ cells per high power field. Definitive IgG4-RD was identified by suggestive organ involvement associated with elevated serum IgG4 levels and histological features (15). For AAV, we used the American College of Rheumatology (ACR) 1990 criteria and the definitions from the 2012 Chapel Hill Consensus Conference and the EMA algorism for GPA, MPA, and EGPA (16–18). Due to a lack of histopathological examination, only a possible diagnosis of IgG4-RD was established in our two patients (Nos. 1, 2) according to the currently accepted criteria (6), although we cannot fully exclude the diagnosis of AAV in the current stage. We diagnosed four cases with definitive IgG4-RD and three with probable and possible Ig4-RD. Eight patients had anti-MPO ANCA, and two had anti-proteinase-3 (PR3) ANCA. Two cases (Nos. 7 and 10) fulfilled the CDC for IgG4-RD and the ACR and Chapel Hill criteria and the EMA algorism for AAV. One had biopsy results compatible with both GPA and IgG4-RD, and the other (No. 7) had a clinical overlap. Eight patients had documented symptom courses, and most of them had a diagnostic delay of several months before admission. In patients with intracranial dura mater involved, headache was the most common symptom, followed by cranial nerve deficits, such as hypoacusis, dysphagia, diplopia, vertigo, hypoglossal nerve palsy, and trigeminal nerve palsy. Headaches were often described as a sense of persistent local crushing with progressive exacerbation and refraction to drugs. Two patients (Nos. 4 and 5) had papilledema, most likely caused by intracranial hypertension. Patients with spinal hypertrophic pachymeningitis often experienced radicular pain and spinal cord compression symptoms (Nos. 3, 6, and 8), but rarely fever. MRI revealed focal pachymeningitis in eight cases, with the posterior fossa and occipital lobe most commonly involved, which might explain the neurological symptoms. Lymphocytosis and oligoclonal bands were commonly found in the CSF, as well as elevated blood non-specific inflammatory indicators, such as CPR and ESR. Eight patients had elevated serum IgG4 levels, while one displayed mild neutrophil-predominant leukocytosis, thrombocytosis, and normocytic anemia. The overall responses to immunosuppressants were good, with six cases attaining great clinical and radiological improvement; two cases suffered from one or two relapses, and only one patient expired from secondary infection.

**Table 1:** Clinical, radiological, and laboratorial features of the patients.

**No**.	**Age and sex**	**Onset**	**Clinical features**	**MRI dural thickening location**	**Serum IgG4 (mg/dL)**	**Findings about AAV**	**ANCA(+) status**	**Pathology**	**IgG4-RD based on CDC (15)**	**Tx**
1	65M	Sub.	Headache, weight loss, dysphagia, diplopia, fever	Posterior fossa	411	No	C, MPO	na	Possible	CTC CTX
2	52F	Sub.	Headache, hypoglossal nerve palsy	Posterior fossa bilateral posterior cerebral	512	No	P, MPO	na	Possible	CTC CTX
3	61F	Chr.	Spinal cord compression symptoms	Spinal dura (T7 - T11)	441	No	P, MPO	IgG4+ cells rich infiltration, fibrosis	Definite	CTC
4. Popkirov et al. (8)	52M	Sub.	Headache, blurred vision, hearing impairment, tinnitus, and vertigo	Infratentorial	NA	No	P, MPO	IgG4+ cells rich infiltration	Probable	CTC AZA RTX
5. Massey et al. (9)	70M	Sub.	Headache, transient visual loss, syncope	Diffuse	233	No	P, MPO	Storiform fibrosis, IgG4+ cells rich infiltration	Definite	CTC
6. Maher et al. (10)	79F	Sub.	Thoracic back pain	Spinal dura (C6–L1)	↑	No	MPO,	Storiform fibrosis, IgG4+ cells rich infiltration, obliterative phlebitis	Definite	CTC RTX
7. Wyrostek et al. (11)	48M	Sub.	Headache, weight loss, hearing impairment, mastoiditis and pansinusitis, proteinuria, lung nodules	Posterior fossa bilateral posterior cerebral hemispheres	245	GPA	C, PR3	Increased IgG4+ cells (lung nodule and bone marrow)	Probable	CTC RTX
8. Cação et al. (12)	56F	Sub.	Lumbar pain, medullary symptoms	Dorsal and lumbar spinal dura	↑	No	MPO	na	Possible	CTC
9. Musto et al. (13)	59F	na	Headache neck pain	Foramen magnum	NA	No	C, PR3	Several IgG4+ cells	Probable	NA
10. Mori et al. (14)	73M	Chr.	Headaches, ophthalmalgia, blurred vision	Diffuse	156	GPA	MPO	Lymphocytes and rich IgG4+ cells infiltration, granulomatous inflammation	Definite	CTC

ANCA, anti-neutrophil cytoplasmic antibody; AZA, azathioprine; C, cytoplasmic; CDC, Comprehensive Diagnostic Criteria; Chr., Chronic; CTC, corticosteroids; CTX, cyclophosphamide; GPA, granulomatosis with polyangiitis; MPO, myeloperoxidase; na, not available; P, perinuclear; PR3, proteinase 3; RTX, rituximab; Sub., subacute; Tx, treatment.

## Discussion

In our pooled analysis, two cases (Nos. 7 and 10) fulfilled the diagnostic criteria for both diseases, while the others only had ANCA seropositivity without additional clinical or pathological markers of AAV. Although IgG4-RD commonly involves multiple organs synchronously or metachronously, the clinical symptoms and radiological features of most patients in this study resulted from the involvement of a single organ (dura mater). They had elevated serum IgG4 levels (>135 mg/dl for most patients) and positive ANCA. Although AAV is an important differential diagnosis of IgG4-RD in both the 2019 ACR/EULAR classification criteria (19) and CDC for IgG4-RD, a recent report concluded that the presence of ANCA might not influence the pathomechanisms of IgG4-RD (20). Furthermore, an overlap of AAV and IgG4-RD has been reported in some clinical patterns, such as tubulointerstitial nephritis, periaortitis, and prevertebral fibrosis (21, 22). A prior case report also suggested that HP caused by GPA and IgG4-RD might be a disorder spectrum (12). However, controversy and counter-examples exist. A study with 62 patients diagnosed with IgG4-RD found no cases with overlapping AAV, challenging the coexistence of IgG4-RD and AAV (23). Meanwhile, our case review of IgG4-HP patients coexpressing ANCA is in line with the existence of the overlap syndrome.

Recently, Martin-Nares et al. (24) reported that IgG4-RD patients coexpressing ANCA often had the following characteristics: (1) constitutional symptoms with salivary glands, lymph nodes, and kidneys involved; (2) high levels of serum total IgG, IgG1, and IgG4; (3) low C3 and C4 levels; (4) high prevalence of antinuclear antibody positivity. The authors interpreted these results as indicating that the ANCA detected in patients with IgG4-RD does not indicate an underlying AAV overlap but might instead represent an excessive B-cell response. However, our case review did not confirm these features in IgG4-HP patients coexpressing ANCA.

Currently, the pathogenesis of AAV and IgG4-RD is not entirely understood. Several studies have confirmed that many patients with EGPA do have elevated blood IgG4 levels. These levels are correlated with disease activity, suggesting that AAV and IgG4-RD have similar or associated pathogeneses. Several studies have shown that, in both disorders, T-follicular helper cell levels increase and become polarized toward T-follicular helper 2 subtype cells, enhancing IgG4-plasma cell polarization (25). Further investigation of the pathogenesis of these disorders may improve the understanding of the IgG4-ANCA coexistence. In addition, the close relation between GPA and IgG4-RD may be partly explained by the fact that, in GPA, ANCA belongs mainly to the IgG1 and IgG4 subclass. Thus, ANCA production could be induced after prolonged or repeated antigen exposures in the setting of a T-helper-2 cell immune response, where T-regulatory cells are activated and produce interleukins 4 and 10, which contribute to the shift of balance of IgG subclasses toward IgG4 (26). However, the pathophysiology of both AAV and IgG4-RD is complex and warrants further research.

Finally, the therapeutic approach remains also important. Current treatment strategies for IgG4-RD include glucocorticoids and conventional steroid-sparing agents, such as low-dose cyclophosphamide and mycophenolate mofetil. Noteworthily, three patients used B-cell depletion therapy (Nos. 4, 6, and 7). Rituximab demonstrated efficacy against both AAV and IgG4-RD (27–29), including in overlapping cases (21). Patients with IgG4-RD may also benefit from 500 mg of rituximab every 6 months for at least 2 years (29), which is the standard approach to maintain clinical remission in AAV cases.

This study has several limitations. First, the inclusion and exclusion criteria in the selection of cases could not be rigorously applied according to the CDC and 2019 ACR/EULAR classification criteria for IgG4-RD, causing inevitable deficiencies in the literature cases. Second, we only performed a pathological examination on one of our cases and, therefore, cannot rule out other pathologies in the other two patients (such as lymphoma, inflammatory myofibroblastic tumor, Rosai Dorfman disease, Castleman's disease, ulcerative colitis, and rheumatoid meningitis). Third, not all patients had documented CSF levels of ANCA and IgG4. Those were increased in some reports of HP related to IgG4-RD (30) and AAV (31), making them valuable biomarkers for differentiating between IgG4-HP and ANCA-related HP.

## Conclusion

In conclusion, we report three cases of IgG4-HP coexpressing ANCA. By analyzing them with previously reported cases, we confirmed the existence of an overlap syndrome for IgG4-RD and AAV in the clinical pattern of HP. Meanwhile, our results do not support the hypothesis that ANCA detected in IgG4-RD results from an excessive B-cell response in HP. Thus, we speculate that IgG4-RD and AAV have similar or associated pathogeneses, although further information is needed about the role of IgG4 and ANCA in their pathophysiological processes. Nevertheless, the question remains: do IgG4-RD and AAV belong to the spectrum of a single disease or simply overlap sometimes?

## Data availability statement

The raw data supporting the conclusions of this article will be made available by the authors, without undue reservation.

## Ethics statement

The studies involving human participants were reviewed and approved by the Medical Ethical Research Committee of General Hospital of Northern Theater Command. The patients/participants provided their written informed consent to participate in this study. Written informed consent was obtained from the individual(s) for the publication of any potentially identifiable images or data included in this article.

## Author contributions

CX: drafting/revision of the manuscript for content, including medical writing for content, major role in the acquisition of data, and analysis or interpretation of data. PL: study concept or design. All authors have read and approved the final manuscript to be published.

## Conflict of interest

The authors declare that the research was conducted in the absence of any commercial or financial relationships that could be construed as a potential conflict of interest.

## Publisher's note

All claims expressed in this article are solely those of the authors and do not necessarily represent those of their affiliated organizations, or those of the publisher, the editors and the reviewers. Any product that may be evaluated in this article, or claim that may be made by its manufacturer, is not guaranteed or endorsed by the publisher.
